# Prognostic Impact of Unplanned Hospitalization During First-Line Gemcitabine Plus Nab-Paclitaxel Therapy for Unresectable Pancreatic Cancer: A Single-Center Retrospective Observational Study

**DOI:** 10.3390/cancers18020194

**Published:** 2026-01-07

**Authors:** Kazuki Watabe, Motoyasu Kan, Izumi Ohno, Sodai Uchida, Taiga Sudo, Koki Yokozuka, Akinori Abe, Yoshiki Nakaya, Yoshiki Ogane, Hiroki Kurosaki, Miho Sakai, Yu Sekine, Tomoya Takahashi, Mayu Ouchi, Hiroshi Ohyama, Nozomu Sakai, Shigetsugu Takano, Tsukasa Takayashiki, Masayuki Ohtsuka, Jun Kato

**Affiliations:** 1 Department of Gastroenterology, Graduate School of Medicine, Chiba University, 1-8-1 Inohana, Chuo-ku, Chiba 260-8670, Japan; 2 Department of Medical Oncology, Graduate School of Medicine, Chiba University, 1-8-1 Inohana, Chuo-ku, Chiba 260-8670, Japan; 3 Department of General Surgery, Graduate School of Medicine, Chiba University, 1-8-1 Inohana, Chuo-ku, Chiba 260-8670, Japan

**Keywords:** pancreatic cancer, gemcitabine plus nab-paclitaxel, unplanned hospitalization, prognostic factor, gemcitabine, nab-paclitaxel, biliary obstruction

## Abstract

Background: Pancreatic cancer (PC) is a refractory malignancy with a dismal prognosis. For unresectable PC, gemcitabine plus nab-paclitaxel (GnP) is widely used as first-line chemotherapy. During treatment, patients may require unplanned hospitalization (UPH) due to tumor progression, biliary obstruction, or chemotherapy-related adverse events. Although UPH during chemotherapy may be linked to poorer survival, its prognostic impact as a time-dependent clinical event during active treatment has not been empirically evaluated in unresectable PC. We investigated the prognostic impact of UPH occurring during first-line GnP therapy. Objective: To clarify the association between UPH during first-line GnP and overall survival (OS). Methods: We retrospectively analyzed 189 patients with histologically confirmed unresectable PC who received first-line GnP at our institution between February 2016 and February 2023. The occurrence of UPH during GnP and the reason for the first UPH were categorized. Associations with OS were assessed using the Kaplan–Meier method and Cox proportional hazards models, including a time-varying covariate (TVC) analysis. Risk factors for UPH were examined with logistic regression. Results: UPH occurred in 76 patients (40.2%) during GnP. Pancreatic head tumors and pre-treatment biliary drainage were significantly more frequent in the UPH group. Median OS was 10.88 months in the UPH group versus 19.23 months in the non-UPH group; UPH was a significant adverse prognostic factor (hazard ratio [HR] 1.97, *p* < 0.01). In multivariable analysis incorporating a TVC, UPH remained an independent predictor of worse prognosis (HR 3.02, *p* < 0.01). Reasons for first UPH were progression (*n* = 28), recurrent biliary obstruction (RBO; *n* = 26), GnP-related adverse event (AE; *n* = 16), and other (*n* = 6). Hospitalization due to progression or RBO was associated with poorer survival. Pancreatic head location was identified as a risk factor for UPH. Conclusions: UPH during first-line GnP is an independent adverse prognostic factor in patients with unresectable PC, even after accounting for TVC. In pancreatic head cancer, closer monitoring for biliary and obstructive complications may be particularly important during treatment.

## Simple Summary

Unresectable pancreatic cancer is usually treated with combination chemotherapy using gemcitabine and nab-paclitaxel. During treatment, some patients suddenly need to be admitted to hospital because of cancer progression, infections, or treatment-related problems. It is unclear how such unplanned hospitalizations affect patient survival in real-world practice. In this single-center study, we reviewed the medical records of patients who started gemcitabine plus nab-paclitaxel as their first treatment for unresectable pancreatic cancer. We compared survival between patients who experienced at least one unplanned hospitalization during treatment and those who did not. We also examined the main reasons for admission. We found that unplanned hospitalization, especially due to cancer progression or recurrent biliary obstruction, was associated with shorter survival. These findings may help clinicians identify vulnerable patients earlier, support clinical decision-making, and optimize supportive care during chemotherapy.

## 1. Introduction

Pancreatic cancer (PC) is a refractory malignancy with a 5-year survival rate of 8.5% in Japan [1]. Most cases are detected at an unresectable stage, and even among resected cases, frequent recurrence remains a major cause of poor prognosis. Although advances in perioperative treatment and palliative chemotherapy are expected to prolong survival [2], cure is still difficult to achieve. Gemcitabine plus nab-paclitaxel (GnP), widely used as first-line therapy, has been reported to yield a median progression-free survival of 5.5 months and a median overall survival (OS) of 8.5 months [3]. Given the dismal prognosis of pancreatic cancer, identifying prognostic factors and translating them into actionable strategies in routine practice is a key clinical priority. Reported adverse prognostic factors in unresectable disease include poor Eastern Cooperative Oncology Group (ECOG) performance status (PS) [4,5], a modified Glasgow Prognostic Score (mGPS) of 2 [6], liver metastasis [7], an elevated neutrophil-to-lymphocyte ratio (NLR) [4,5], and high or rising levels of carbohydrate antigen 19-9 (CA19-9) during treatment [8]. However, most of these predictors are baseline characteristics assessed before treatment initiation, and the impact of events occurring during therapy has not been sufficiently investigated.

Unplanned hospitalization (UPH) during systemic chemotherapy may reflect a complex interplay of disease progression, treatment-related toxicity, and patient vulnerability. Importantly, UPH has the potential to disrupt treatment continuity through dose reduction, treatment delay, or premature discontinuation, which may be particularly consequential in pancreatic cancer, where maintaining treatment intensity is critical for survival benefit.

Pancreatic cancer is more often diagnosed following an emergency presentation than other malignancies, with reported rates of 50–66.7%; such pre-diagnostic emergency department (ED) visits are associated with worse outcomes, partly through lower odds of initiating treatment [9,10]. Among eight major cancers (esophagus, stomach, colon, rectum, liver, pancreas, lung, ovary), pancreatic cancer has the highest proportion of diagnoses made within 30 days of an emergency admission (46.1%), and emergency presentation is a strong predictor of 12-month mortality (adjusted odds ratio > 2.5) [11]. These observations indicate that ED presentation or hospitalization before diagnosis is an adverse prognostic factor. Moreover, in hepatobiliary–pancreatic tumors, the rate of experiencing at least one unplanned hospitalization (UPH) within one year after diagnosis is as high as 57.9% [12]. In pancreatic cancer specifically, hospitalization within 14 days after initiating first-line GnP occurs in 16.4% of patients [13], and over the disease course the rates of ED visits and UPH have been reported as 77.7% and 59.3%, respectively [14]. Nevertheless, most prior studies have focused on emergency presentation or hospitalization occurring before diagnosis or treatment initiation, while the prognostic impact of UPH arising during active chemotherapy remains insufficiently characterized. In particular, few studies have evaluated UPH as a time-dependent clinical event or examined its impact according to the underlying reasons for hospitalization. Unlike many baseline prognostic factors, unplanned hospitalization may serve as a clinically observable on-treatment marker reflecting disease aggressiveness, treatment-related toxicity, and patient vulnerability. Clarifying its prognostic impact may therefore help identify high-risk patients who could benefit from closer surveillance or timely supportive care during chemotherapy. Therefore, in this study, we investigated the prognostic impact of unplanned hospitalization during first-line GnP therapy in patients with unresectable pancreatic cancer, with particular attention to the timing and causes of hospitalization.

## 2. Materials and Methods

### 2.1. Study Design and Patients

This was a single-center, retrospective observational study. We identified 343 consecutive patients who were histologically diagnosed with pancreatic cancer and initiated systemic chemotherapy at our institution between February 2016 and February 2023. Exclusion criteria were: (i) first-line therapy other than GnP; (ii) treatment primarily delivered at another hospital (or an indeterminate treatment history at our hospital); (iii) resectable or borderline-resectable disease; and (iv) discontinuation of chemotherapy before completing one cycle (early termination). After applying these criteria, 189 patients with unresectable pancreatic cancer who started first-line GnP were included in the analysis.

### 2.2. Data Collection

From the electronic medical records, we extracted clinical data including patient demographics and baseline characteristics, treatment details, occurrence and causes of UPH, and survival outcomes. For patients who underwent biliary drainage, available information on drainage modality (plastic biliary stent, self-expandable metal stent (SEMS), endoscopic ultrasound-guided drainage (EUS-BD), percutaneous transhepatic biliary drainage (PTBD), or other drainage) and planned stent exchange strategies was collected when available. Decisions regarding elective stent exchange were made at the discretion of the treating physician, and no standardized institutional protocol was applied. Planned elective stent exchanges were not considered UPH.

### 2.3. Definitions

Unplanned hospitalization (UPH): An unplanned admission occurring during the GnP treatment period, triggered by symptom onset that prompted an outpatient visit and led to hospitalization within a few days. Hospitalizations before GnP initiation or after GnP cessation, and planned admissions solely for chemotherapy administration, were excluded.

#### Classification of the First UPH Cause

- Progression: UPH prompted by new biliary or duodenal obstruction or by symptoms attributable to disease progression.
- Recurrent biliary obstruction (RBO): UPH for cholangitis or biliary obstruction in patients who had undergone biliary drainage before treatment; RBO was defined in accordance with the TOKYO criteria 2024 [15].
- Adverse events (AE): UPH for conditions or symptoms that could not rule out attribution to GnP.
- Other: UPH for conditions judged clinically to have little direct relation to pancreatic cancer or GnP and not meeting the above definitions (Progression, RBO, AE).

When multiple categories could apply, priority was assigned in the order: Progression > RBO > AE > Other. This prioritization rule was predefined to ensure consistent classification across cases and was applied uniformly throughout the study. Causes of admission were adjudicated based on chart review, laboratory/imaging findings, and, when relevant, microbiological results. In brief, biliary obstruction was classified as progression when accompanied by radiologic evidence of tumor growth or new obstructive lesions, whereas RBO was assigned when obstruction or cholangitis occurred in previously drained ducts without clear radiologic progression, in accordance with the TOKYO criteria 2024. The first author performed the initial adjudication, which was verified by a senior physician; discrepancies were resolved by discussion. No formal standardized adjudication algorithm or independent dual review process was implemented beyond this predefined framework. Neither independent dual adjudication nor inter-rater agreement (κ) was assessed. Admissions attributed to AE were classified according to clinical causality (suspected relationship to GnP), not by CTCAE grade; infections with an identified pathogen and considered unlikely to be related to GnP were classified as Other.

### 2.4. Endpoints

Primary endpoint: The impact of UPH during GnP on OS, defined as the time from GnP initiation to death from any cause or censored at the date last known alive. Secondary endpoints: OS according to the cause of the first UPH, and risk factors for the occurrence of UPH. Cause-specific UPH was evaluated descriptively using Kaplan–Meier analyses; multivariable or time-varying covariate modeling stratified by UPH cause was not performed due to limited sample size within each subgroup.

### 2.5. Handling of Cut-Off Values for Clinical Variables

**Figure A1:**
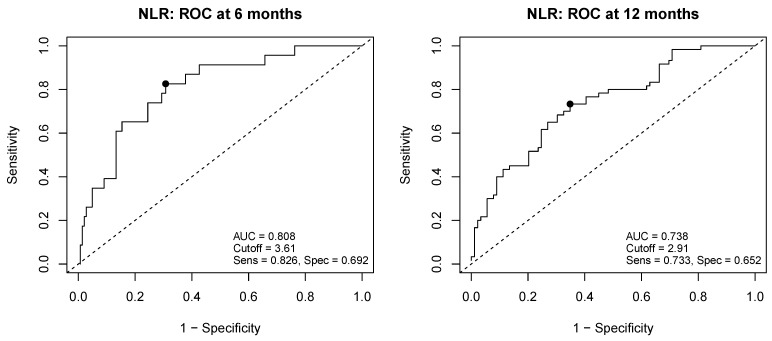
ROC curves of baseline NLR for predicting mortality at 6 and 12 months. The dashed diagonal line indicates no discrimination, and the black dot indicates the optimal cutoff determined by Youden’s index.

Albumin (Alb), C-reactive protein (CRP), and NLR were evaluated as markers of systemic inflammation and nutritional status. Cut-offs for CRP and Alb were set according to our institutional upper limits of normal: CRP > 1.0 mg/dL and Alb < 3.0 g/dL. NLR was calculated as absolute neutrophils/lymphocytes. Using receiver operating characteristic (ROC) analysis with the 6-month outcome after treatment initiation (death vs. alive) as the endpoint—and excluding cases censored before 6 months—we identified in an exploratory manner the Youden index-maximizing cut-off of 3.61 for the main analyses (Figure A1). As a sensitivity analysis, we repeated the procedure using the 12-month outcome and derived a cut-off of 2.91; results using NLR 2.91 are presented in Table A1. The CRP and albumin cut-offs were based on institutional reference values and were used for pragmatic clinical categorization rather than to define biologically or prognostically optimal thresholds.

### 2.6. Statistical Analysis

Descriptive statistics summarized baseline characteristics. Continuous variables are presented as medians [interquartile range] and compared between groups using the Mann–Whitney U test. Categorical variables were compared using the $\chi^{2}$ test or Fisher’s exact test, as appropriate. OS was estimated by the Kaplan–Meier method, and groups were compared using the log-rank test. Prognostic factors were evaluated using Cox proportional hazards models; effect sizes are reported as hazard ratios (HRs) with 95% confidence intervals (CIs). UPH was incorporated as a time-varying covariate (TVC) in an extended Cox model to perform multivariable analyses accounting for time dependence. This approach was chosen to mitigate potential immortal time bias by allowing patients to contribute risk time appropriately before and after the occurrence of UPH. Only variables with *p* < 0.05 in univariable analyses were entered into multivariable models. Risk factors for UPH were assessed using logistic regression, with results expressed as odds ratios (ORs) and 95% CIs. Model calibration was evaluated with the Hosmer–Lemeshow goodness-of-fit test using standard grouping, and discrimination with the area under the ROC curve (AUC); 95% CIs for AUCs were obtained by DeLong’s method. Multicollinearity was assessed using the variance inflation factor (VIF), with VIF < 5 indicating acceptable collinearity. For both regression and survival analyses, complete-case analysis was employed; cases with missing values for covariates were excluded from the corresponding analyses (no imputation). Two-sided α was set at 0.05. *p*-values were reported to two decimal places; values < 0.01 were presented as *p* < 0.01. Analyses were conducted using R version 4.5.1 (R Foundation for Statistical Computing, Vienna, Austria) and RStudio version 2025.05.1 (Posit Software, Boston, MA, USA, PBC), primarily with the survival package (and survminer when needed). Detailed longitudinal data on relative dose intensity and dose modifications were not uniformly available for all patients and were therefore not included in the present analyses.

### 2.7. Ethics

This retrospective study was approved by the Institutional Review Board of Chiba University Hospital (approval No. 3317; initial approval: 23 January 2019; renewal upon continuing review: 30 April 2025). Study information was posted on the hospital website, and informed consent was obtained via an opt-out process. The study was conducted in accordance with the Declaration of Helsinki and relevant guidelines.

## 3. Results

### 3.1. Patient Selection

**Figure 1:**
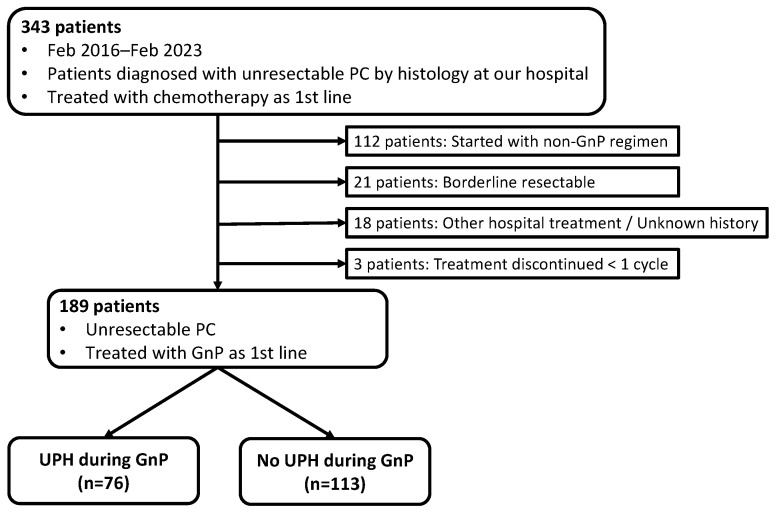
Selection of patients for the study. PC: pancreatic cancer, GnP: gemcitabine plus nab-paclitaxel, UPH: unplanned hospitalization. Between February 2016 and February 2023, 343 patients with histologically confirmed pancreatic cancer who initiated systemic chemotherapy at our institution were screened. After exclusion of patients who received first-line regimens other than GnP, had borderline-resectable disease, received treatment primarily at another institution, or discontinued chemotherapy before completing one cycle, 189 patients with unresectable pancreatic cancer treated with first-line GnP were included in the final analysis. Patients were classified according to the occurrence of UPH during GnP therapy into the UPH group (*n* = 76) and the no UPH group (*n* = 113).

Between February 2016 and February 2023, we identified 343 patients at our institution who had a histologic diagnosis of pancreatic cancer (PC), were deemed unresectable by imaging, and initiated systemic chemotherapy. We excluded 112 patients who started a first-line regimen other than GnP, 21 with borderline-resectable disease, 18 who received treatment primarily at another hospital or had an indeterminate treatment history at our hospital, and 3 who discontinued therapy before completing one cycle. The final cohort comprised 189 patients with unresectable PC who began first-line GnP. Patients were classified into two groups according to the occurrence of UPH during GnP: UPH (*n* = 76) and no UPH (*n* = 113) (Figure 1).

### 3.2. Baseline Characteristics

Baseline characteristics are summarized in Table 1. UPH occurred in 76 patients and did not occur in 113 patients. Pancreatic head tumors were more frequent in the UPH group than in the no UPH group (50/76 [65.8%] vs. 36/113 [31.9%]; *p* < 0.01). A history of pre-treatment biliary drainage was also more common in the UPH group (32/76 [42.1%] vs. 18/113 [15.9%]; *p* < 0.01). No significant differences were observed for other baseline variables. ECOG PS ≥ 1 was numerically more frequent in the UPH group than in the no UPH group (26.3% vs. 15.9%), although this difference did not reach statistical significance (*p* = 0.12). Among patients who underwent pre-treatment biliary drainage, plastic biliary stents were more frequently used in the UPH group than in the no UPH group (25/76 [32.9%] vs. 8/113 [7.1%] of the overall cohort; *p* < 0.01). Other drainage consisted of two patients: one who underwent surgical gastrojejunostomy with biliary-enteric anastomosis for gastrointestinal obstruction complicated by retrograde cholangitis, and one who underwent endoscopic stone extraction for choledocholithiasis without biliary stenting. The distribution of non-plastic biliary drainage modalities did not differ markedly between the UPH and no UPH groups, although the number of patients for each modality was limited. Reasons for the first UPH were: progression in 28 patients (36.8%), RBO in 26 (34.2%), GnP-related AE in 16 (21.1%), and other causes in 6 (7.9%) (Table 1). Among AE-related admissions (*n* = 16), the causes were: infection associated with neutropenia in 6 (bacterial pneumonia, *n* = 2; pulmonary aspergillosis, *n* = 1; calculous cholecystitis, *n* = 1; sepsis, *n* = 2), febrile neutropenia in 4, nausea/anorexia in 3, heart failure in 1 (in the context of diarrhea and anemia), fever in 1 (suspected drug or tumor fever), and hypoglycemia in 1. “Other” causes (*n* = 6) included depression, microbiologically confirmed Salmonella gastroenteritis, osteoporotic vertebral compression fracture, syncope, otolaryngologic disorder (hemorrhage from vestibular schwannoma) and retrograde cholangitis after gastrojejunostomy (one case each).

### 3.3. Survival by UPH Status

**Figure 2:**
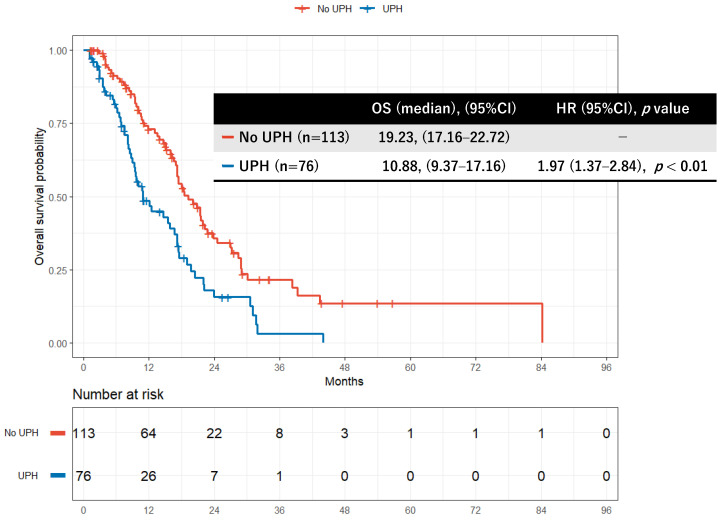
Kaplan–Meier curve comparing overall survival between UPH group and no UPH group during GnP. GnP: gemcitabine plus nab-paclitaxel, UPH: unplanned hospitalization. Kaplan–Meier curves show overall survival for patients with unresectable pancreatic cancer treated with first-line GnP, stratified by the occurrence of UPH during treatment. Median overall survival was 19.23 months (95% CI, 17.16–22.16) in the no UPH group and 10.88 months (95% CI, 9.37–17.16) in the UPH group. Survival was significantly worse in patients who experienced UPH (log-rank *p* < 0.01).

Kaplan–Meier curves for OS according to UPH status are shown in Figure 2. Median OS was 19.23 months (95% CI, 17.16–22.16) in the no UPH group and 10.88 months (95% CI, 9.37–17.16) in the UPH group (log-rank *p* < 0.01). UPH during GnP was associated with significantly worse survival (HR, 1.97; 95% CI, 1.37–2.84; *p* < 0.01) (Figure 2).

### 3.4. Prognostic Factor Analysis

Results of Cox proportional hazards analyses are presented in Table 2. In univariable analyses, male sex was a favorable prognostic factor, whereas PS ≥ 1, liver metastasis, Alb < 3.0 g/dL, CRP > 1.0 mg/dL, NLR ≥ 3.61, and UPH during first-line GnP were adverse factors. Pre-treatment biliary drainage showed borderline statistical significance in univariable analysis (*p* = 0.07) and was included in the multivariable models given its clinical relevance and close association with UPH. In the multivariable model without TVC, male sex remained a favorable prognostic factor, whereas liver metastasis, CRP > 1.0 mg/dL, NLR ≥ 3.61, and UPH remained adverse prognostic factors. In the TVC-adjusted multivariable model, the same variables remained significant, and ECOG PS ≥ 1 showed borderline significance (*p* = 0.05). The HR for UPH was 2.34 (95% CI, 1.55–3.56; *p* < 0.01) in the conventional multivariable model and 3.02 (95% CI, 2.00–4.56; *p* < 0.01) in the TVC-adjusted model (Table 2).

### 3.5. Survival by Reason for UPH

**Figure 3:**
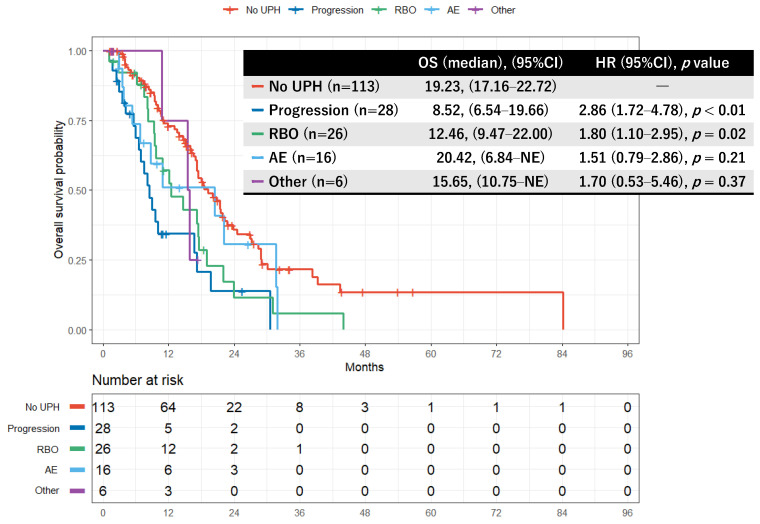
Kaplan–Meier curves comparing overall survival between the no UPH group and groups classified by the reason for first UPH. GnP: gemcitabine plus nab-paclitaxel; UPH: unplanned hospitalization; NE: not estimable; RBO: recurrent biliary obstruction; AE: adverse events. Reasons for UPH were categorized as follows: Progression, UPH due to new biliary or duodenal obstruction or symptoms attributable to disease progression; RBO, UPH due to cholangitis or biliary obstruction after pre-treatment biliary drainage; AE, UPH for conditions possibly related to GnP therapy; Other, UPH for causes not meeting the above definitions. Kaplan–Meier curves compare overall survival among patients without UPH and those experiencing a first UPH due to disease progression, RBO, treatment-related AE, or other causes during first-line GnP therapy. Median overall survival was 19.23 months (95% CI, 17.16–22.16) in the no UPH group; 8.52 months (95% CI, 6.54–19.66) in the progression group; 12.46 months (95% CI, 9.47–22.00) in the RBO group; 20.42 months (95% CI, 6.54–not estimable) in the AE group; and 15.65 months (95% CI, 15.65–not estimable) in the Other group. Overall differences among groups were statistically significant (log-rank *p* < 0.01). Compared with the no UPH group, survival was significantly worse in patients hospitalized for progression or RBO.

Kaplan–Meier curves comparing the no UPH group with each first-UPH category are shown in Figure 3. The median OS was 19.23 months (95% CI, 17.16–22.16) in the no UPH group; 8.52 months (95% CI, 6.54–19.66) in the progression group; 12.46 months (95% CI, 9.47–22.00) in the RBO group; 20.42 months (95% CI, 6.54–not estimable [NE]) in the AE group; and 15.65 months (95% CI, 15.65–NE) in the Other group. Overall differences among groups were significant (*p* < 0.01). Compared with the no UPH group, HRs were 2.86 (95% CI, 1.72–4.78; *p* < 0.01) for progression and 1.80 (95% CI, 1.10–2.95; *p* = 0.02) for RBO, indicating significantly worse survival. The HRs for the AE group (1.51; 95% CI, 0.79–2.86; *p* = 0.21) and the Other group (1.70; 95% CI, 0.53–5.46; *p* = 0.37) did not reach statistical significance but trended toward worse outcomes (Figure 3).

### 3.6. Risk Factors for UPH

To identify factors associated with UPH, we performed logistic regression with UPH as the dependent variable (Table 3). In univariable analyses, pancreatic head location, Alb < 3.0 g/dL, CRP > 1.0 mg/dL, and a history of pre-treatment biliary drainage were significant risk factors. In multivariable analysis including the significant univariable covariates, only pancreatic head location remained an independent risk factor for UPH. Model calibration was adequate (Hosmer–Lemeshow $\chi^{2}$ = 4.69, df = 5, *p* = 0.454), and discrimination was acceptable (AUC = 0.73; 95% CI, 0.66–0.80). Multicollinearity was low (variance inflation factor, 1.08–1.33).

## 4. Discussion

### 4.1. Main Findings

To our knowledge, this is the first retrospective cohort study to examine the prognostic impact of UPH during first-line GnP therapy in patients with unresectable PC. Among 189 patients, 76 (40.2%) experienced UPH during first-line GnP. UPH was consistently associated with shorter OS in univariable and multivariable Cox models, and remained an independent adverse prognostic factor when modeled as a TVC. The most frequent initial cause of UPH was progression (*n* = 28), followed by RBO (*n* = 26), AE related to GnP (*n* = 16), and other causes (*n* = 6). Cause-specific analyses showed that hospitalizations for progression and RBO were significantly associated with worse prognosis. Pancreatic head location was identified as a risk factor for UPH. UPH may reflect an underlying adverse clinical trajectory characterized by declines in PS and activities of daily living (ADLs), as well as interruptions or delays in chemotherapy, rather than acting as a direct causal factor. In this context, UPH may be viewed as an on-treatment dynamic clinical marker capturing disease aggressiveness and patient vulnerability not fully reflected by baseline characteristics. The relatively favorable OS observed in the AE-related UPH group should be interpreted with caution, as this subgroup was small and may represent patients who were able to continue treatment after transient toxicities or who had relatively preserved baseline characteristics. Prior tumor-agnostic studies have demonstrated that declines in ADLs and instrumental ADLs (IADLs) during chemotherapy correlate strongly with shorter survival [16,17]. In male patients with PC, on-treatment loss of subcutaneous fat mass has been reported as an independent adverse prognostic factor [18]. In addition, patients with unresectable PC often experience worsening quality of life (QOL) during chemotherapy, characterized by increased adverse events and heightened anxiety about the future [19]. These phenomena can co-occur with UPH and provide a plausible pathway linking UPH to poor survival. In our cohort, progression accounted for the largest share of UPH (36.8%), suggesting that UPH can be a clinical manifestation of advancing disease. Some RBO events likely reflect stent occlusion driven by tumor progression; moreover, cholangitis during chemotherapy may contribute to poor outcomes through declines in PS/ADLs. The relatively high proportion of plastic biliary stent use in our cohort may partly reflect institutional and referral-related factors. Specifically, a subset of patients were referred with plastic biliary stents already placed at outside hospitals, and at our institution, plastic biliary stents are commonly selected until a histological diagnosis is established, after which conversion to SEMS may be considered. Notably, biliary drainage is required in approximately 60.0% of patients with unresectable PC, underscoring biliary obstruction as a major complication [20]. In pancreatic head cancer, cholangitis during neoadjuvant therapy has been associated with treatment delays and inferior OS (HR 2.06; 95% CI, 1.08–3.91) [21]. Sepsis accounts for 32–49.7% of intensive care unit (ICU) admissions among patients with PC, roughly half due to biliary infections, and outcomes after ICU admission are extremely poor [22,23]. By contrast, in our study, AE-related UPH during GnP was not significantly associated with prognosis. Prior reports suggest that grade ≥ 3 hematologic toxicities during chemotherapy may correlate with better outcomes in unresectable PC [24], implying that AE-related admissions do not necessarily portend an immediate adverse prognosis.

### 4.2. Clinical Implications of Risk Factors for UPH

Median OS with GnP in Japanese cohorts has been reported as 17.1 months [25], comparable to outcomes in our study. The slightly favorable survival observed in our cohort may partly reflect institutional practice patterns and patient selection, including the exclusion of patients who discontinued therapy before completing one treatment cycle. Reported risk factors for UPH at the time of PC diagnosis include jaundice, ECOG PS ≥ 2–4, age ≥ 75 years, and thrombophlebitis [26]. Tumor-agnostic cohorts have identified low serum albumin, hyponatremia, ADL impairment, and abnormal geriatric 8 (G8) screening tool scores as predictors of hospitalization during treatment [27,28,29]. In our cohort, low albumin, impaired PS, and age were not associated with UPH; instead, pancreatic head location emerged as the key risk factor. Anatomically, head tumors more readily cause biliary or duodenal obstruction, leading to earlier symptom manifestation with disease progression, and patients are also susceptible to post-drainage complications. Indeed, in unresectable pancreatic head cancer, biliary stricture and duodenal stenosis have been reported in 81% and 25% of cases, respectively [30]. Notably, although ECOG PS showed only borderline significance in the conventional multivariable model, its prognostic impact became more evident after incorporating UPH as a time-varying covariate, suggesting that baseline functional status and dynamic on-treatment events independently contribute to prognosis.

### 4.3. Potential Strategies to Mitigate UPH

Beyond its association with survival, UPH poses additional challenges. Physical activity restrictions during hospitalization may accelerate sarcopenia; longer hospital stays can directly worsen QOL both physically and psychologically. Recurrent UPH can limit employment and impose financial and caregiving burdens. Practical measures may help reduce potentially preventable hospitalizations during chemotherapy. In patients receiving modified FOLFIRINOX (folinic acid [leucovorin], fluorouracil [5-FU], irinotecan, and oxaliplatin), the presence of a biliary stent has been linked to shorter OS (median, 10.3 vs. 24.9 months) and higher rates of biliary events and febrile neutropenia [31]. During chemotherapy for PC, preventive and early interventions for biliary events—and initial selection of self-expandable metal stents—have been associated with fewer UPHs and treatment interruptions [32]. Early drainage has also shown benefit in those requiring percutaneous transhepatic biliary drainage (PTBD) [33]. Given that 75.3% of UPHs in hepatobiliary–pancreatic cancers present through the emergency department [12], routine electronic patient-reported outcome (ePRO) monitoring with timely outreach [34] and early palliative care integration soon after diagnosis [35] have been linked to fewer ED visits. Collectively, these data are consistent with a potential role for early oncologic and supportive-care interventions in reducing the risk of UPH.

### 4.4. Limitations

This study has limitations. It is a single-center, retrospective analysis without a priori sample-size calculation. Decisions to hospitalize were at the discretion of treating physicians, and no uniform institutional criteria were enforced. Accordingly, admission thresholds, biliary drainage strategies, and supportive-care practices may differ across institutions, which may limit the generalizability of our findings to other clinical settings.

AE-related admissions were adjudicated by a single reviewer with senior verification; neither independent dual adjudication nor inter-rater reliability (κ) was assessed. Misclassification is therefore possible; however, we adopted conservative rules (e.g., categorizing microbiologically confirmed infections with low likelihood of GnP causality as Other) to mitigate bias. Despite these efforts, misclassification between progression-related biliary obstruction and recurrent biliary obstruction cannot be completely excluded, particularly in cases with overlapping clinical features. In addition, the definition of UPH relied on clinical judgment documented in medical records, and variability among physicians or over time cannot be ruled out. Excluding direct emergency admissions and not stratifying hospitalizations by length of stay or severity may have resulted in heterogeneous clinical events being grouped together. We also did not perform stratified analyses according to tumor location (pancreatic head vs. body–tail). Given the close anatomical and clinical relationship between pancreatic head tumors, biliary drainage, and biliary events, biliary drainage may function as a mediator rather than an independent confounder; however, the limited sample size precluded robust stratified modeling. Moreover, analyses of time-dependent events such as UPH are susceptible to immortal time bias if not appropriately modeled. To mitigate this, we incorporated UPH as a time-varying covariate in extended Cox models, allowing patients to contribute risk time appropriately before and after the occurrence of UPH. In addition, detailed longitudinal data on relative dose intensity and dose modifications were not uniformly available and were therefore not incorporated into the analyses, which may have influenced both the occurrence of UPH and survival outcomes. Additionally, patients who discontinued GnP therapy before completing one treatment cycle were excluded, which may have preferentially removed the sickest patients and introduced selection bias. This exclusion was applied to ensure consistent exposure to first-line GnP when evaluating treatment-related UPH. Finally, we did not directly test whether preventing UPH improves survival; prospective studies are needed to establish causality. Despite these limitations, our study provides the first evidence that UPH during first-line GnP is an independent adverse prognostic factor even after accounting for time dependence, and it offers practical implications supporting vigilant biliary management and early supportive interventions. Notably, GnP-related AE admissions constituted only 21.1% of UPHs in this cohort, suggesting that most UPHs derive from oncologic and biliary complications rather than regimen-specific toxicities—an observation that may generalize to other treatment regimens.

### 4.5. Clinical Implications

Because patients who experience UPH are at higher risk of poor outcomes, timely transition to subsequent therapy and early use of social resources and palliative care may help maximize treatment benefit and shorten hospital stays. In pancreatic head cancer, stringent monitoring for biliary or duodenal obstruction and cholangitis is essential. Selecting stents with longer patency and considering planned exchanges may further optimize biliary management and may be considered as part of efforts to reduce potentially preventable UPH.

## 5. Conclusions

Unplanned hospitalization during first-line gemcitabine plus nab-paclitaxel therapy is a significant adverse prognostic factor in patients with unresectable pancreatic cancer. By explicitly accounting for its time-dependent nature, this study demonstrates that unplanned hospitalization functions as an independent and dynamic prognostic indicator during treatment. Incorporating unplanned hospitalization into routine clinical assessment may facilitate early identification of high-risk patients and support timely optimization of treatment intensity, supportive care, and patient counseling.

## Figures and Tables

**Table 1:** Baseline patients’ characteristics and reasons for first UPH during first-line GnP.

	All (*n* = 189)	UPH During GnP (*n* = 76)	No UPH During GnP (*n* = 113)	*p* Value
Age (years)				
Median [IQR]	68.0 [62.0–74.0]	68.0 [62.0–74.0]	68.5 [63.0–75.0]	0.69
Sex				
Male, *n* (%)	102 (54.0%)	43 (56.6%)	59 (52.2%)	0.66
Female, *n* (%)	87 (46.0%)	33 (43.4%)	54 (47.8%)	
ECOG PS				
≥1, *n* (%)	38 (20.1%)	20 (26.3%)	18 (15.9%)	0.12
0, *n* (%)	151 (79.9%)	56 (73.7%)	95 (84.1%)	
Tumor Location				
Head, *n* (%)	86 (45.5%)	50 (65.8%)	36 (31.9%)	<0.01
Body–Tail, *n* (%)	103 (54.5%)	26 (34.2%)	77 (68.1%)	
Depth of invasion				
T4, *n* (%)	114 (60.3%)	44 (57.9%)	70 (61.9%)	0.68
T3≥, *n* (%)	75 (39.7%)	32 (42.1%)	43 (38.1%)	
Disease extension				
Metastatic, *n* (%)	132 (69.8%)	52 (68.4%)	80 (70.8%)	0.85
Locally advanced, *n* (%)	57 (30.2%)	24 (31.6%)	33 (29.2%)	
Metastatic site				
Liver, *n* (%)	90 (47.6%)	36 (47.4%)	54 (47.8%)	1.00
Lung, *n* (%)	25 (13.2%)	11 (14.5%)	14 (12.4%)	0.84
Peritoneal, *n* (%)	28 (14.8%)	9 (11.8%)	19 (16.8%)	0.46
Lymph node, *n* (%)	47 (24.9%)	21 (27.6%)	26 (23.0%)	0.58
Pre-treatment biliary drainage, *n* (%)	50 (26.5%)	32 (42.1%)	18 (15.9%)	<0.01
with Plastic biliary stent, *n* (%)	33 (17.5%)	25 (32.9%)	8 (7.1%)	<0.01
with SEMS, *n* (%)	11 (5.8%)	2 (2.6%)	9 (8.0%)	0.20
with EUS-BD, *n* (%)	3 (1.6%)	2 (2.6%)	1 (0.9%)	0.57
with PTBD, *n* (%)	1 (0.5%)	1 (1.3%)	0 (0.0%)	0.40
with Other drainage, *n* (%)	2 (1.1%)	2 (2.6%)	0 (0.0%)	0.16
History of other cancer, *n* (%)	37 (19.6%)	17 (22.4%)	20 (17.7%)	0.54
Family history of PC, *n* (%)	18 (9.5%)	7 (9.2%)	11 (9.7%)	1.00
Reason for first UPH (within UPH group)				
Progression, *n* (%)		28 (36.8%)		
RBO, *n* (%)		26 (34.2%)		
AE, *n* (%)		16 (21.1%)		
Other, *n* (%)		6 (7.9%)		
Other (*n* = 6), one case each:				
– Depression				
– Microbiologically confirmed Salmonella gastroenteritis				
– Osteoporotic compression fracture				
– Syncope				
– Hemorrhage from a vestibular schwannoma				
– Reflux cholangitis after gastrojejunostomy				

IQR: interquartile range, UPH: unplanned hospitalization, GnP: Gemcitabine + nab-Paclitaxel, ECOG PS: Eastern Cooperative Oncology Group Performance Status, SEMS: self-expandable metal stent, EUS-BD: endoscopic ultrasound-guided biliary drainage, PTBD: percutaneous transhepatic biliary drainage, RBO: recurrent biliary obstruction, AE: adverse event, PC: pancreatic cancer. “with x” indicates pre-treatment biliary drainage performed using modality x. “Other drainage” consisted of two patients: one who underwent surgical gastrojejunostomy with biliary-enteric anastomosis for gastrointestinal obstruction and retrograde cholangitis, and one who underwent endoscopic stone extraction for choledocholithiasis without biliary stenting. “Reason for first UPH” is summarized only for the UPH subgroup. AE admissions were classified by clinical causality with GnP rather than CTCAE grading. AE-related UPH (*n* = 16) comprised neutropenia-associated infections in 6 cases (bacterial pneumonia, *n* = 2; pulmonary aspergillosis, *n* = 1; calculous cholecystitis, *n* = 1; sepsis, *n* = 2), febrile neutropenia, *n* = 4; nausea/anorexia, *n* = 3; heart failure, *n* = 1 (following diarrhea and anemia); fever, *n* = 1 (suspected drug- or tumor-related); and hypoglycemia, *n* = 1.

**Table 2:** Univariate and multivariate analyses including UPH during GnP.

Variable	Univariate			Multivariate Without TVC			Multivariate with TVC		
	HR	95% CI	*p* Value	HR	95% CI	*p* Value	HR	95% CI	*p* Value
Age									
<70 (reference)	1.00								
≥70	0.89	0.62–1.29	0.54						
Sex									
female (reference)	1.00								
male	0.61	0.43–0.88	<0.01	0.62	0.43–0.89	0.01	0.63	0.44–0.91	0.01
ECOG PS									
0 (reference)	1.00								
≥1	1.67	1.08–2.58	0.02	1.57	1.00–2.47	0.05	1.72	1.17–2.51	<0.01
Tumor location									
Body–tail (reference)	1.00								
Head	1.35	0.94–1.94	0.10						
Depth of invasion									
T3 ≥ (reference)	1.00								
T4	0.94	0.65–1.36	0.74						
Disease extension									
Locally advanced (reference)	1.00								
Metastatic	1.01	0.69–1.48	0.96						
Metastatic site									
None (reference)	1.00								
Liver	2.02	1.40–2.92	<0.01	2.04	1.37–3.03	<0.01	2.12	1.40–3.22	<0.01
Lung	0.90	0.52–1.58	0.72						
Peritoneal	0.85	0.51–1.43	0.55						
Lymph node	1.29	0.84–1.98	0.24						
Albumin (g/dL)									
≥3.0 (reference)	1.00								
<3.0	2.95	1.46–5.96	<0.01	1.36	0.64–2.93	0.43	1.29	0.63–2.64	0.49
CRP (mg/dL)									
≤1.0 (reference)	1.00								
>1.0	3.03	1.99–4.61	<0.01	1.89	1.17–3.06	<0.01	1.72	1.01–2.92	0.04
CEA (ng/mL)									
≤4.8 (reference)	1.00								
>4.8	1.08	0.76–1.56	0.66						
CA19-9 (U/mL)									
≤35.4 (reference)	1.00								
>35.4	1.06	0.67–1.69	0.79						
NLR									
<3.61 (reference)	1.00								
≥3.61	2.66	1.82–3.88	<0.01	2.38	1.57–3.61	<0.01	2.37	1.64–3.43	<0.01
Pre-treatment biliary drainage									
No (reference)	1.00								
Yes	1.44	0.97–2.14	0.07	0.94	0.60–1.47	0.79	0.84	0.56–1.25	0.39
UPH during GnP									
No (reference)	1.00								
Yes	1.97	1.37–2.84	<0.01	2.34	1.55–3.56	<0.01	3.02	2.00–4.56	<0.01

For UPH, a multivariable analysis accounting for a time-varying covariate (TVC) was performed. UPH: unplanned hospitalization, GnP: gemcitabine plus nab-paclitaxel, HR: hazard ratio, CI: confidence interval, ECOG PS: Eastern Cooperative Oncology Group performance status, CRP: C-reactive protein, CEA: carcinoembryonic antigen, CA19-9: carbohydrate antigen, NLR: neutrophil-to-lymphocyte ratio. Variables not included in the multivariable models were omitted because they showed no association with overall survival in univariable analyses (*p* ≥ 0.05). Pre-treatment biliary drainage was included in the multivariable models despite borderline statistical significance in univariable analysis (*p* = 0.07), given its clinical relevance and close association with UPH and biliary complications.

**Table 3:** Risk factors for UPH during GnP therapy.

Variable	Univariate Analysis			Multivariate Analysis		
	OR	95% CI	*p* Value	OR	95% CI	*p* Value
Age						
<70 (reference)	1.00					
≥70	1.02	0.57–1.83	0.95			
Sex						
female (reference)	1.00					
male	1.19	0.67–2.15	0.55			
ECOG PS						
0 (reference)	1.00					
≥1	1.88	0.92–3.89	0.08			
Tumor location						
Body–tail (reference)	1.00					
Head	4.11	2.24–7.72	<0.01	3.18	1.52–6.65	<0.01
Depth of invasion						
T3 ≥ (reference)	1.00					
T4	0.84	0.47–1.53	0.58			
Disease extension						
Locally advanced (reference)	1.00					
Metastatic	0.89	0.48–1.69	0.73			
Metastatic site						
None (reference)	1.00					
Liver	0.98	0.55–1.76	0.95			
Lung	1.20	0.51–2.80	0.68			
Peritoneal	0.66	0.28–1.56	0.35			
Lymph node	1.28	0.66–2.49	0.47			
Albumin (g/dL)						
≥3.0 (reference)	1.00					
<3.0	3.27	1.11–10.89	0.04	2.29	0.64–8.11	0.20
CRP (mg/dL)						
≤1.0 (reference)	1.00					
>1.0	2.15	1.07–4.34	0.03	1.79	0.81–3.92	0.15
CEA (ng/mL)						
≤4.8 (reference)	1.00					
>4.8	0.74	0.41–1.33	0.31			
CA19-9 (U/mL)						
≤35.4 (reference)	1.00					
>35.4	0.72	0.33–1.56	0.40			
NLR						
<3.61 (reference)	1.00					
≥3.61	1.60	0.88–2.90	0.12			
Pre-treatment biliary drainage						
No (reference)	1.00					
Yes	3.57	1.86–7.02	<0.01	1.75	0.77–3.99	0.18

OR: odds ratio, CI: confidence interval, ECOG PS: Eastern Cooperative Oncology Group performance status, CRP: C-reactive protein, CEA: carcinoembryonic antigen, CA19-9: carbohydrate antigen, GnP: gemcitabine plus nab-paclitaxel, UPH: unplanned hospitalization, NLR: neutrophil-to-lymphocyte ratio.

## Data Availability

The data supporting this study are available from the corresponding author upon reasonable request.

## Abbreviations

The following abbreviations are used in this manuscript:

PC	Pancreatic cancer
GnP	Gemcitabine plus nab-paclitaxel
UPH	Unplanned hospitalization
OS	Overall survival
TVC	Time-varying covariate
HR	Hazard ratio
RBO	Recurrent biliary obstruction
AE	Adverse event
ECOG	Eastern cooperative oncology group
PS	Performance status
mGPS	modified Glasgow Prognostic Score
NLR	Neutrophil-to-lymphocyte ratio
CA19-9	Carbohydrate antigen 19-9
ED	Emergency department
Alb	Albumin
CRP	C-reactive protein
ROC	Receiver operating characteristic
CI	Confidence interval
OR	Odds ratio
AUC	Area under the ROC curve
VIF	Variance inflation factor
IQR	Interquartile range
SD	Standard deviation
CEA	Carcinoembryonic antigen
SEMS	Self-expandable metal stent
EUS-BD	Endoscopic ultrasound-guided biliary drainage
PTBD	Percutaneous transhepatic biliary drainage
NE	Not estimable
ADLs	Activities of daily living
IADLs	Instrumental ADLs
QOL	Quality of life
ICU	Intensive care unit
G8	Geriatric 8
FORFIRINOX	folinic acid (leucovorin), fluorouracil (5-FU), irinotecan, and oxaliplatin
ePRO	electronic patient-reported outcome

## Appendix A

### Appendix A.1. Determination of the Optimal NLR Cutoff Values Using Time-Dependent ROC Analysis

The NLR optimal cutoff (Youden) was 3.61 at 6 months (AUC = 0.81, 95% CI 0.712–0.893; sensitivity 0.826, specificity 0.692) and 2.91 at 12 months (AUC = 0.74; sensitivity 0.733, specificity 0.652). Outcomes were dichotomized at each time point (death before t vs. alive at t); patients censored before t were excluded from that analysis (Figure A1).

### Appendix A.2. Sensitivity Analyses Using an Alternative NLR Cutoff (12-Month ROC–Derived Value)

Sensitivity analyses of Cox proportional hazards models using the alternative NLR cutoff of 2.91, derived from a 12-month dichotomized ROC analysis (Youden’s index). Results are presented as univariate, multivariable (without TVC), and multivariable (with TVC). The estimates were directionally and quantitatively consistent with the main analysis using the 3.61 cutoff (6-month ROC), and high NLR (≥2.91) remained an independent adverse prognostic factor (Table A1).

**Table A1:** Univariate and multivariate analyses including UPH during GnP (set NLR cutoff to 2.91).

Variable	Univariate			Multivariate Without TVC			Multivariate with TVC		
	HR	95% CI	*p* Value	HR	95% CI	*p* Value	HR	95% CI	*p* Value
Age									
<70 (reference)	1.00								
≥70	0.89	0.62–1.29	0.54						
Sex									
female (reference)	1.00								
male	0.61	0.43–0.88	<0.01	0.63	0.44–0.92	0.02	0.64	0.44–0.94	0.02
ECOG PS									
0 (reference)	1.00								
≥1	1.67	1.08–2.58	0.02	1.60	1.02–2.51	0.04	1.74	1.20–2.52	<0.01
Tumor location									
Body–tail (reference)	1.00								
Head	1.35	0.94–1.94	0.10						
Depth of invasion									
T3 ≥ (reference)	1.00								
T4	0.94	0.65–1.36	0.74						
Disease extension									
Locally advanced (reference)	1.00								
Metastatic	1.01	0.69–1.48	0.96						
Metastatic site									
None (reference)	1.00								
Liver	2.02	1.40–2.92	<0.01	1.95	1.31–2.90	<0.01	2.01	1.32–3.06	<0.01
Lung	0.90	0.52–1.58	0.72						
Peritoneal	0.85	0.51–1.43	0.55						
Lymph node	1.29	0.84–1.98	0.24						
Albumin (g/dL)									
≥3.0 (reference)	1.00								
<3.0	2.95	1.46–5.96	<0.01	1.32	0.61–2.85	0.48	1.20	0.59–2.47	0.61
CRP (mg/dL)									
≤1.0 (reference)	1.00								
>1.0	3.03	1.99–4.61	<0.01	2.28	1.42–3.67	<0.01	2.10	1.26–3.50	<0.01
CEA (ng/mL)									
≤4.8 (reference)	1.00								
>4.8	1.08	0.76–1.56	0.66						
CA19-9 (U/mL)									
≤35.4 (reference)	1.00								
>35.4	1.06	0.67–1.69	0.79						
NLR									
<2.91 (reference)	1.00								
≥2.91	2.04	1.42–2.93	<0.01	1.58	1.07–2.33	0.02	1.58	1.08–2.30	0.02
Pre-treatment biliary drainage									
No (reference)	1.00								
Yes	1.44	0.97–2.14	0.07	0.95	0.61–1.49	0.82	0.86	0.59–1.27	0.45
UPH during GnP									
No (reference)	1.00								
Yes	1.97	1.37–2.84	<0.01	2.06	1.37–3.09	<0.01	2.75	1.82–4.15	<0.01

For UPH, a multivariable analysis accounting for a time-varying covariate (TVC) was performed. UPH: unplanned hospitalization, GnP: gemcitabine plus nab-paclitaxel, HR: hazard ratio, CI: confidence interval, ECOG PS: Eastern Cooperative Oncology Group performance status, CRP: C-reactive protein, CEA: carcinoembryonic antigen, CA19-9: carbohydrate antigen. Variables not included in the multivariable models were omitted because they showed no association with overall survival in univariable analyses (*p* ≥ 0.10). Pre-treatment biliary drainage was included in the multivariable models despite borderline statistical significance in univariable analysis (*p* = 0.07), given its clinical relevance and close association with UPH and biliary complications.
